# Cerebrolysin as an Early Add-on to Reperfusion Therapy: Risk of Hemorrhagic Transformation after Ischemic Stroke (CEREHETIS), a prospective, randomized, multicenter pilot study

**DOI:** 10.1186/s12883-023-03159-w

**Published:** 2023-03-27

**Authors:** Dina R. Khasanova, Mikhail N. Kalinin

**Affiliations:** 1 Department of Neurology and Neurosurgery for Postgraduate Training, Kazan State Medical University, Kazan, Russia; 2 Department of Neurology, Interregional Clinical Diagnostic Center, 12A Karbyshev St, Kazan, 420101 Russia

**Keywords:** Cerebrolysin, Hemorrhagic transformation, Stroke, Reperfusion therapy

## Abstract

**Background:**

Cerebrolysin could mitigate reperfusion injury and hemorrhagic transformation (HT) in animal models of acute ischemic stroke.

**Methods:**

This was a prospective, randomized, open-label, parallel-group with active control, multicenter pilot study. Cerebrolysin (30 mL/day over 14 days) was administered concurrently with alteplase (0.9 mg/kg) in 126 patients, whereas 215 control patients received alteplase alone. The primary outcomes were the rate of any and symptomatic HT assessed from day 0 to 14. The secondary endpoints were drug safety and functional outcome measured with the National Institutes of Health Stroke Scale (NIHSS) on day 1 and 14, and the modified Rankin scale (mRS) on day 90. Advanced brain imaging analysis was applied on day 1 and 14 as a marker for in vivo pharmacology of Cerebrolysin.

**Results:**

Cerebrolysin treatment resulted in a substantial decrease of the symptomatic HT rate with an odds ratio (OR) of 0.248 (95% CI: 0.072–0.851; *p* = 0.019). No serious adverse events attributed to Cerebrolysin occurred. On day 14, the Cerebrolysin arm showed a significant decrease in the NIHSS score (*p* = 0.045). However, no difference in the mRS score was observed on day 90. A substantial improvement in the advanced brain imaging parameters of the infarcted area was evident in the Cerebrolysin group on day 14.

**Conclusions:**

Early add-on of Cerebrolysin to reperfusion therapy was safe and significantly decreased the rate of symptomatic HT as well as early neurological deficit. No effect on day 90 functional outcome was detected. Improvements in the imaging metrics support the neuroprotective and blood–brain barrier stabilizing activity of Cerebrolysin.

**Trial registration:**

Name of Registry: ISRCTN.

Trial Registration Number: ISRCTN87656744.

Trial Registration Date: 16/02/2021.

## Introduction

Intravenous thrombolysis (IVT) within 4.5 h after acute ischemic stroke (AIS) substantially improves functional outcome [1]. However, complications of IVT such as reperfusion injury and hemorrhagic transformation (HT) might significantly aggravate stroke-related morbidity and mortality. Neuroprotection is considered as a strategy to mitigate those adverse consequences.

A plethora of experimental stroke models have demonstrated neuroprotective effects of Cerebrolysin and its ability to attenuate blood–brain barrier (BBB) permeability [2, 3]. Several clinical trials and meta-analyses have suggested Cerebrolysin enhances early post-stroke recovery and improves neurological deficit after AIS [4–6].

In our current study, we looked at the effects of Cerebrolysin with IVT versus IVT alone in AIS patients. The rationale behind our research comes from two aspects. First, recombinant tissue plasminogen activator (rtPA) increases the HT rate by degrading the BBB integrity, and promoting neuroinflammation and excitotoxicity [7–9]. On the other hand, Cerebrolysin ameliorates rtPA adverse effects and, therefore, can potentially protect from rtPA-related HT [3, 7, 10]. Thus, the combination of the two agents could be beneficial for AIS patients.

Our primary goal was the assessment of the HT rate. The secondary objectives included the evaluation of treatment safety and functional outcome. In addition, advanced brain imaging analysis was applied in an attempt to demonstrate in vivo the neuroprotective and BBB stabilizing activity of Cerebrolysin.

## Methods

CEREHETIS was a prospective, randomized, open-label, active control, multicenter, parallel-group phase IIIb pilot study. The patients were enrolled across 8 centers in Russia – the Interregional Clinical Diagnostic Center (Kazan), Municipal Clinical Hospital #7 (Kazan), Kazan Federal University Hospital (Kazan), Perm Territorial Clinical Hospital (Perm), Emergency Medical Center (Naberezhnye Chelny), Leninogorsk District Hospital (Leninogorsk), Nizhnekamsk District Hospital (Nizhnekamsk), and Arsk District Hospital (Arsk) – from April 2018 to August 2020.

### Inclusion and exclusion criteria

Main inclusion and exclusion criteria are outlined in Table 1.

**Table 1:** Inclusion and exclusion criteria

**Inclusion criteria:** • Confirmed diagnosis of acute ischemic stroke • Age ≥ 18 years • Onset of stroke symptoms within 4.5 h before initiation of rtPA administration
**Exclusion criteria:** • Current or previous intracranial hemorrhage • Symptoms suggestive of subarachnoid hemorrhage, even if CT scan was normal • Imaging data on admission suggestive of a brain tumor, arteriovenous malformation, brain abscess or intracerebral aneurism • Previous history of brain tumor, intracranial aneurism or arteriovenous malformation • Previous history of brain or spine surgery • Acute myocardial infarction within the previous 3 months • Major bleeding, current or within the previous 6 months • Gastrointestinal or genitourinary bleeding within the previous 3 months • Confirmed relapse of gastric or duodenal ulcer • Unknown time of symptom onset • Minor (NIHSS score < 4) or severe stroke (NIHSS score > 25) on admission • Seizure at stroke onset • Stroke or serious head trauma within the previous 3 months • Administration of heparin within the 48 h preceding the stroke onset, with an activated partial thromboplastin time at presentation exceeding the upper limit of the normal range • Platelet count < 100 × 10^9^/L • Systolic blood pressure > 185 mm Hg or diastolic blood pressure > 110 mm Hg • Blood glucose < 50 mg/dL (2.8 mmol/L) or > 400 mg/dL (22.2 mmol/L) • Oral anticoagulant treatment • Major surgery or severe trauma within the previous 3 months • Other major disorders associated with an increased risk of bleeding (neoplasm, bleeding diathesis, acute pancreatitis, infective endocarditis, pericarditis, severe liver and kidney failure) • Known allergic reactions to rtPA, Cerebrolysin and its components • Pregnancy and lactation • Endovascular treatment

All recruited patients admitted to the Interregional Clinical Diagnostic Center (Kazan, Russia) who met additional inclusion criteria were consecutively subjected to advanced brain imaging. The criteria were as following: no contraindications to magnetic resonance imaging (MRI) and computed tomography (CT) perfusion (CTP) study, AIS in the middle cerebral artery territory with a minimum diffusion-weighted imaging (DWI) lesion diameter of 20 mm on admission. Patients with any HT on a follow-up CT scan were excluded from the analysis due to paramagnetic distortions caused by blood on the diffusion-tensor imaging (DTI).

The center was selected for advanced brain imaging because it was the only one with MRI being available 24/7.

### Randomization and blinding

Each eligible patient was randomly assigned into either the Cerebrolysin or control group by simple randomization procedure. One randomization list for all centers was issued by generating Bernoulli variates with the probability parameter of 0.333. The Mersenne twister was used as an active generator and the starting point was set at random.

Allocation instructions were sealed in opaque envelopes, mixed and distributed between the centers by an independent statistician. Each envelop was randomly picked by the investigators and was opened after the subject’s recruitment.

Investigators enrolled participants, assigned them to the intervention, and assessed clinically the primary and secondary endpoints. Imaging data were evaluated locally by radiologists who were blinded to the intervention. However, investigators and participants were not blinded to the treatment assignment because Cerebrolysin had its particular yellowish color and we were not able to conceal it properly.

### Study treatment

Both groups received a standard dose of 0.9 mg/kg rtPA (alteplase) administered intravenously within 4.5 h after symptom onset (maximal dosage 90 mg, 10% of the drug given in bolus and the rest in 60 min via intravenous infusion). In addition, measures of standard care for AIS patients were applied for both groups. Patients in the Cerebrolysin group additionally received 30 mL of Cerebrolysin diluted in 100 mL of normal saline administered intravenously through a separate line over 20 min. Cerebrolysin treatment was initiated simultaneously with IVT and continued once daily for 14 consecutive days. Acceptable and prohibited co-medications are listed in Table 2.

**Table 2:** List of Acceptable and Prohibited Co-Medications

**Acceptable Co-Medications:** • Baseline stroke therapy • Medications for compensation of electrolyte and acid–base abnormalities • Symptomatic medications (antihypertensive, antidiabetic agents, drugs to normalize sleep (excluding benzodiazepines), antibiotics, and antipyretics)
**Prohibited Co-Medications:** • Neuroprotective or nootropic agents (citicoline, memantine, amantadine, erythropoietin, diazepam, investigational neuroprotective drugs, piracetam, pramiracetam, pyritinol, meclosulfonate, glycine, etc.) • Medications with vasodilatory effect (naftidrofuryl, cinnarizine, flunarizine, nimodipine, nicergoline, pentoxifylline, ergoloid, vinpocetine, vincamine, ginkgo biloba, etc.) • Antioxidant agents (lipoic acid, ethylmethylhydroxypyridine succinate, etc.) • Levodopa and dopamine agonists • Statins within the first 7 days from the stroke onset

All participants were treated in the hospital settings since inpatient length of stay was at least 14 days according to the national insurance standard for patients with AIS.

Patients were withdrawn from further intervention in case neurosurgery was performed or a life-threatening medical (non-neurological) condition occurred. The patient’s participation was discontinued in case of death. The subjects had the opportunity to exit the study at any time.

The intention-to-treat (ITT) population comprised all recruited patients, subjects completed the study were included in the per-protocol (PP) analysis. The study ended once the required number of patients was reached and the protocol was accomplished by the participants.

### Study procedures

At the time of admission (day 0), the screening and baseline assessment was performed. Routine clinical, laboratory, and imaging data were collected. Follow-up visits were scheduled in 24 h (day 1, visit 1), on day 7 (visit 2), 14 (visit 3) and 90 (visit 4).

The National Institutes of Health Stroke Scale (NIHSS) score was recorded at baseline, in 24 h and on day 14. The modified Rankin scale (mRS) score was assessed on day 90. Vital signs and laboratory tests were evaluated on day 0 and 14.

The Alberta stroke program early CT score (ASPECTS) was assessed at admission. A follow-up CT scan was obtained on day 1, 7 and 14 or at any time if required by a clinician.

The investigators had special training in the NIHSS, mRS, and ASPECTS rating to improve the inter-rater agreement.

### Outcome measures

The study primary endpoints were any and symptomatic HT verified on a follow-up CT scan from day 0 to day 14. Symptomatic HT was defined according to the ECASS III trial: any apparently extravascular blood in the brain or within the cranium that was associated with clinical deterioration, as defined by an increase of 4 points or more in the score on the NIHSS, or that led to death and that was identified as the predominant cause of the neurologic deterioration [1].

Secondary endpoints were the functional outcome measured with the NIHSS and mRS as well as drug safety. Favorable functional outcome was defined as the mRS score of ≤ 2 on day 90. The NIHSS score on day 14 was considered as a marker of short-term neurological recovery [6].

From day 0 to 14, patients in both arms were monitored for any adverse events (AE), including changes in vital signs, general and neurological condition, electrocardiogram, and routine laboratory tests (liver and kidney function tests, complete blood count). In case an AE occurred, the decision to withdraw participants from the study was bestowed upon investigators.

### Advanced brain imaging procedures

On day 1 and 14, a routine brain MRI was acquired followed by an axial DTI scan. The MRI exam was performed on a GE Signa HDx 1.5 T scanner (GE Healthcare, USA). The DTI sequence parameters were as follows: spin-echo echo-planar imaging, repetition time = 6000 ms, echo time = 102.9 ms, field of view = 260 mm, b-value = 0 and 1000 s/mm^2^, matrix = 256 × 256; slice thickness = 4.5 mm, interslice gap = 1 mm, total number of slices = 24, diffusion directions = 25, scan time = 5 min 10 s.

The maps of fractional anisotropy (FA), axial (AD), radial (RD) and mean (MD) diffusivity were derived from the raw DTI scans using the DTIMap plugin for Horos (v.1.6) [11]. On b = 0 image, the threshold was set at 50.

On day 14, a brain CTP scan was obtained using the Dankbaar’s approach [12]. It involves a cine mode CT acquisition, with a temporal sampling rate of one image every 2 s for the first 60 s. Additional gantry rotations were performed at 90, 120, 150, 180, 210 and 240 s. Acquisition parameters were 80 kVp and 100 mA. A bolus of 40 mL iohexol (Omnipaque, GE Healthcare, USA; 300 mg/mL of iodine) was injected into an antecubital vein at an injection rate of 5 mL/s. CT scanning was initiated 5 s after start of the injection of the contrast bolus. A series of CT scans covered the whole brain was obtained with 5 mm slice thickness. The study was performed on a GE Revolution 512-slice CT scanner (GE Healthcare, USA).

The CTP data were processed on a GE Advanced Workstation 4.7 (GE Healthcare, USA) using the CT Perfusion 4D program. As a result, a series of the permeability–surface area product (PS) maps were obtained.

**Fig. 1:**
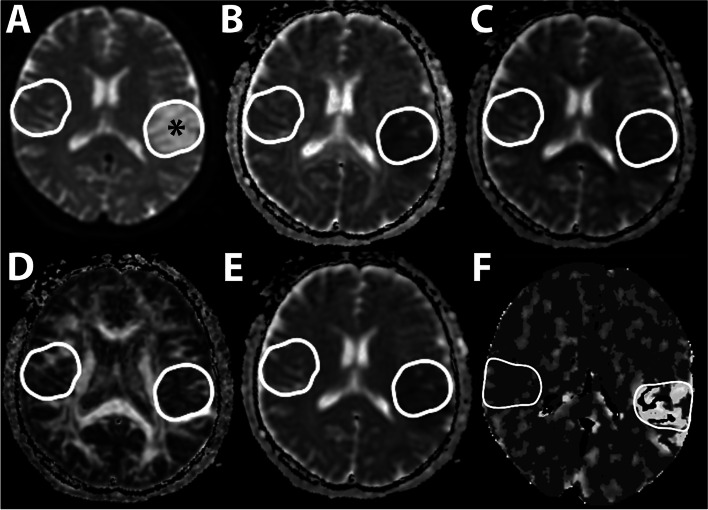
Advanced brain imaging. The most representative slice was selected from a set of diffusion-tensor imaging and CT perfusion scans. The region of interest is outlined on the affected side (*) and is mirrored to the contralateral hemisphere. **A.** Raw DTI scan, b-value = 0 s/mm^2^. **B.** Axial diffusivity map. **C.** Radial diffusivity map. **D.** Fractional anisotropy map. **E.** Mean diffusivity map. **F.** Permeability–surface area product map

The most representative slice was chosen for analysis in each set of images. On that slice, the infarcted area was outlined and mirrored to the contralateral hemisphere. The values of FA, AD, RD, MD, and PS were assessed within each region of interest (Fig. 1).

To cope with the heterogeneity of the ischemic lesions due to different locations, we calculated absolute values of the laterality index for each parameter using the formula [13]: Laterality index= |(Affected side – Unaffected side) / (Affected side + Unaffected side)| × 100%.

The infarct volume was calculated on DWI (on day 1) and CT (on day 14) scans according to the ABC/2 method [14].

### Statistical analysis

Sample size calculation was performed by means of power analysis for matched case–control studies [15]. The minimum detectable odds ratio (OR) for any HT was assumed as low as 0.5. The probability of any HT among controls was expected as high as 0.2 [16]. The significance level, power, and correlation of any HT between the arms were set at the values recommended by the software manual, which were 0.05, 0.8, and 0.3, respectively [15]. As it was a pilot study, any HT was chosen for the calculation because it encompassed all types of HT. The drop-out rate was expected as low as 0.1. A 1:2 design was chosen to reduce the sample size by approximately 30%. Thus, the minimum number of patients in the Cerebrolysin and control arms comprised 88 and 176, respectively.

The estimated number of subjects needed for the advanced brain imaging analysis, computed by power analysis for a two-sample means test [15], was 34 (17 per group). The expected difference in means between the two arms was set as twofold.

Once the desired sample size of 264 participants had been achieved, it became clear that the number of patients included in the advanced brain imaging analysis was insufficient – only 26 patients were recruited. This was due to the additional, more stringent inclusion criteria for the imaging cohort. Thus, we decided to increase the total sample size by issuing additional envelops in the described above manner until that number reached 34.

The descriptive statistics included median (M) with the interquartile range (IQR) for non-normally distributed continuous data and percentage for categorical data.

Groups were compared with the Mann–Whitney U test and Pearson’s χ^2^-test for continuous and categorical variables, respectively.

Odds ratio (OR) was calculated with binary logistic regression followed by* p*-value adjustment using the Romano–Wolf multiple hypothesis correction method with 1,000 bootstrap replications [17]. The number needed to treat (NNT) and its 95% confidence intervals (CI) were calculated by using the Altman procedure and the Daly approach, respectively [18, 19].

The STATA v.14.2 (StataCorp, USA) and IBM SPSS Statistics v.26 (IBM Corporation, USA) software packages were used for statistical analysis.

## Results

**Fig. 2:**
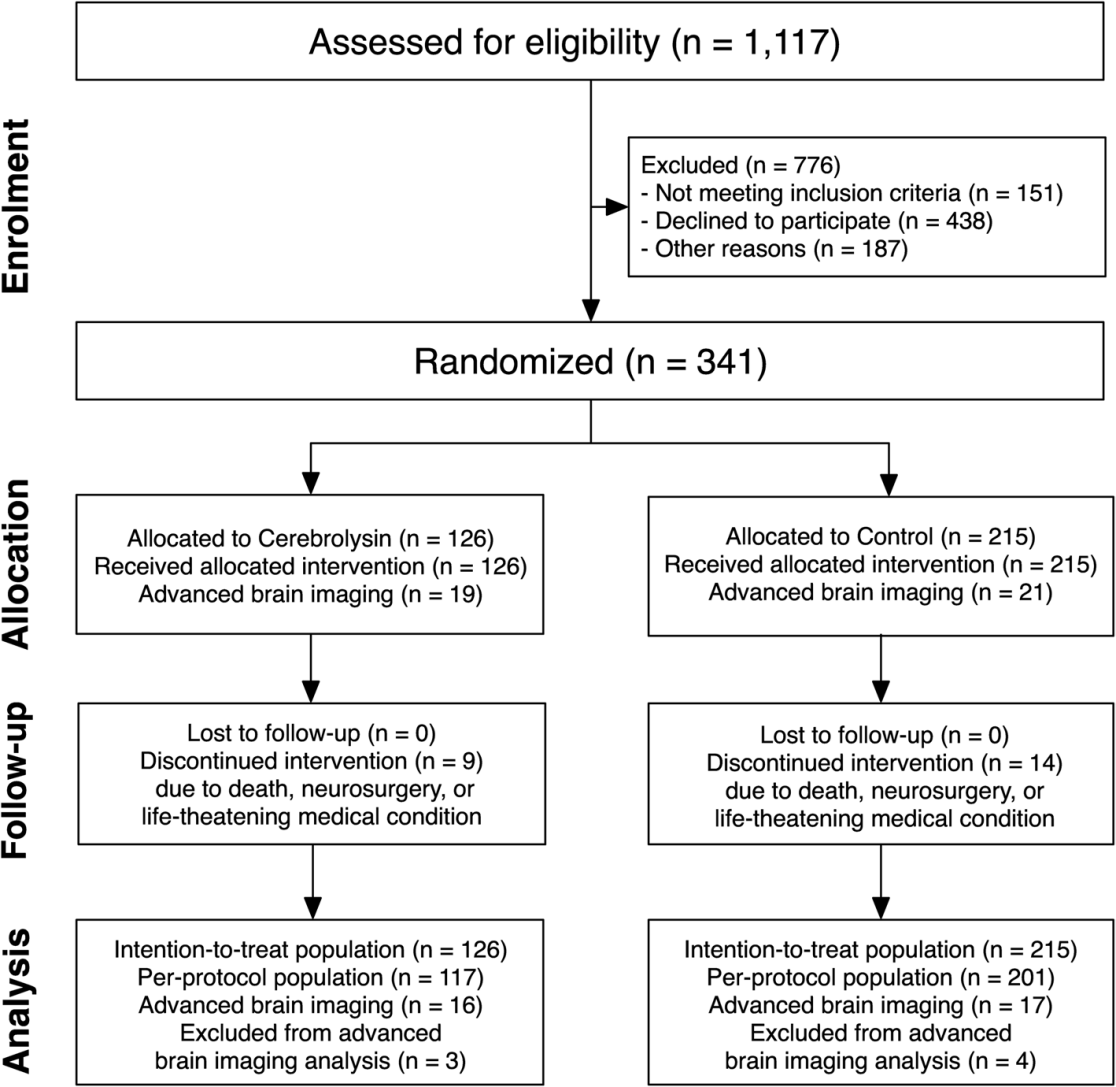
CONSORT flow chart

Of 1,117 assessed patients with AIS who were eligible for IVT, 341 subjects were recruited and constituted the ITT population. Twenty-three participants (6.7%) did not complete the study with the dropout rate being equal between the groups. Thus, 318 patients formed the dataset for PP analysis (Fig. 2).

Although the majority of clinical, laboratory and imaging data were not different between the arms at baseline, the participants occurred to be imbalance in a few variables: the patients in the Cerebrolysin group were slightly younger and, as a result, had fewer cases of hypertension and previous stroke (Table 3).

**Table 3:** Baseline Characteristics

	**ITT Population, ** ***n*** ** = 341**			**PP Population, ** ***n*** ** = 318**		
	**Cerebrolysin** **, ** ***n*** ** = 126**	**Control, ** ***n*** ** = 215**	***p*** **-Value**	**Cerebrolysin** **, ** ***n*** ** = 117**	**Control, ** ***n*** ** = 201**	***p*** **-Value**
Age, yr (M, IQR)	63.5 (56–71)	68 (60–77)	0.003	63 (55.5–70.5)	67 (60–75.5)	0.003
NIHSS at admission (M, IQR)	10 (6–14)	10 (7–14)	0.567	9 (6–13)	9 (6–14)	0.512
ASPECTS at admission (M, IQR)	10 (10–10)	10 (10–10)	0.380	10 (9.5–10)	10 (10–10)	0.285
Atrial fibrillation, *n* (%)	32 (25.4)	42 (19.5)	0.205	29 (24.8)	34 (17.2)	0.103
Diabetes mellitus, *n* (%)	22 (17.5)	32 (14.9)	0.529	18 (15.8)	31 (15.7)	0.975
Sex, male, *n* (%)	76 (60.3)	118 (54.9)	0.328	71 (60.7)	111 (55.2)	0.343
Weight, kg (M, IQR)	80 (68–90)	76 (67–87)	0.380	80 (68–90.5)	75 (66–88.5)	0.359
Systolic blood pressure, mm Hg (M, IQR)	150 (133–163)	150 (140–164)	0.196	149 (130–160.5)	150 (140–163.5)	0.086
Diastolic blood pressure, mm Hg (M, IQR)	90 (80–100)	90 (80–97)	0.621	90 (80–95)	90 (80–92.5)	0.702
Previous use of aspirin or antiplatelet agents, *n* (%)	32 (25.4)	51 (23.7)	0.728	31 (26.5)	55 (27.4)	0.867
Hypertension, *n* (%)	103 (81.7)	193 (89.8)	0.035	94 (80.3)	180 (89.6)	0.022
History of stroke, *n* (%)	16 (12.7)	48 (22.3)	0.028	14 (12.1)	44 (22.6)	0.022
Onset time, min (M, IQR)	105 (80–150)	100 (70–140)	0.199	117.5 (75–150)	97 (70–142.5)	0.134
Door-to-needle time, min (M, IQR)	40 (30–60)	40 (30–65)	0.437	40 (30–60)	40 (30–68)	0.362
Vascular territory, *n* (%)						
Anterior circulation	93 (73.8)	147 (68.4)	0.288	86 (73.5)	134 (66.7)	0.203
Posterior circulation	32 (25.4)	66 (30.7)	0.296	31 (26.5)	65 (32.3)	0.274
Unknown	1 (0.8)	2 (0.9)	0.896	0 (0)	2 (1)	0.279
Stroke subtype, *n* (%)						
Atherothrombotic	39 (31)	74 (34.4)	0.512	36 (30.8)	73 (36.3)	0.315
Cardioembolic	35 (27.8)	63 (29.3)	0.764	32 (27.4)	54 (26.9)	0.925
Lacunar	5 (4)	14 (6.5)	0.323	5 (4.3)	14 (7.0)	0.329
Other known etiology	1 (0.8)	3 (1.4)	0.618	1 (0.9)	3 (1.5)	0.623
Unknown etiology	46 (36.5)	61 (28.4)	0.118	43 (36.8)	57 (28.4)	0.120
Length of inpatient stay, days (M, IQR)	14 (14–14)	14 (14–14)	0.093	14 (14–14)	14 (14–14)	0.066
Discontinue study, *n* (%)	9 (7.1)	14 (6.5)	0.822			

Moreover, the univariate logistic regression analysis revealed a specific set of HT predictors in the recruited patients on admission. Thus, the patients with a higher NIHSS score and serum levels of creatinine and urea were more likely to develop symptomatic HT. In contrast, participants with higher diastolic blood pressure and values of albumin and hemoglobin were less likely to encounter symptomatic intracranial hemorrhage. Interestingly, the well-established risk factors like age, atrial fibrillation, hypertension, diabetes mellitus, previous stroke, and ASPECTS score were not identified as HT predictors in our patients (Table 4).

**Table 4:** Risk factors of symptomatic HT on admission, ITT population, univariate logistic regression, *n* = 341

	**Symptomatic HT, ** ***n*** ** = 24**	**No symptomatic HT, ** ***n*** ** = 317**	**OR (95% CI)**	***p*** **-Value**
Age, y, M (IQR)	70 (63–80)	66 (58–74)	1.036 (1.000–1.074)	0.052
Sex, male, *n* (%)	13 (54.2)	181 (57.1)	0.888 (0.386–2.043)	0.780
NIHSS, M (IQR)	19 (16–20)	9 (6–13)	1.395 (1.247–1.559)	< 0.001
Atrial fibrillation, *n* (%)	8 (33.3)	66 (20.8)	1.902 (0.780–4.635)	0.157
Hypertension, *n* (%)	22 (91.7)	274 (86.4)	1.726 (0.392–7.605)	0.471
Atherosclerosis, *n* (%)	16 (66.7)	178 (56.2)	1.562 (0.650–3.755)	0.319
Diabetes mellitus, *n* (%)	3 (12.5)	51 (16.1)	0.745 (0.214–2.591)	0.644
Previous stroke, *n* (%)	7 (29.2)	57 (18)	1.878 (0.744–4.740)	0.182
Pre-admission antiplatelet agent use, *n* (%)	9 (37.5)	82 (25.9)	1.720 (0.725–4.079)	0.219
Systolic blood pressure, mm Hg, M (IQR)	140 (127–169)	150 (140–163)	0.990 (0.971–1.009)	0.307
Diastolic blood pressure, mm Hg, M (IQR)	85 (78–90)	90 (80–100)	0.959 (0.925–0.994)	0.022
ASPECTS, M (IQR)	10 (10–10)	10 (10–10)	1.109 (0.662–1.858)	0.696
Hyperdense middle cerebral artery sign, *n* (%)	1 (4.2)	11 (3.5)	1.209 (0.150–9.784)	0.858
Albumin, g/L, M (IQR)	41 (36–44)	42 (39–45)	0.889 (0.806–0.980)	0.018
Creatinine, μmol/L, M (IQR)	95 (71–138)	85 (73–101)	1.013 (1.002–1.024)	0.016
Glucose, mmol/L, M (IQR)	7.55 (5.55–8.32)	6.4 (5.5–7.8)	0.999 (0.980–1.017)	0.878
Hemoglobin, g/L, M (IQR)	124 (115–147)	141 (127–149)	0.980 (0.961–0.999)	0.043
Platelet count, × 10^9^/L, M (IQR)	185 (163–219)	218 (179–264)	0.993 (0.986–1.000)	0.058
Hematocrit, M (IQR)	0.38 (0.35–0.43)	0.4 (0.37–0.43)	0.007 (0.000–12.695)	0.196
Urea, mmol/L, M (IQR)	7.36 (3.91–9.7)	5.6 (4.5–7.2)	1.207 (1.034–1.408)	0.017

### Primary endpoints

In both arms, HT occurred mostly within 24 h after IVT. In the ITT population, Cerebrolysin set a favorable trend to lower any HT with a rate of 15.9% versus 23.3% in the control group and a corresponding OR of 0.543 (95% CI: 0.281–1.05; *p* = 0.078). That tendency was even more evident in the PP group, where Cerebrolysin significantly reduced any HT with a rate of 13.7% versus 22.9% in the control group and a corresponding OR of 0.417 (95% CI: 0.200–0.871; *p* = 0.032) (Table 5).

**Table 5:** Study endpoints

	**ITT Population, ** ***n*** ** = 341**			**PP Population, ** ***n*** ** = 318**		
	**Cerebrolysin** **, ** ***n*** ** = 126**	**Control, ** ***n*** ** = 215**	***p*** **-Value**	**Cerebrolysin** **, ** ***n*** ** = 117**	**Control, ** ***n*** ** = 201**	***p*** **-Value**
**Primary**						
HT, *n* (%)						
Any	20 (15.9)	50 (23.3)	0.103	16 (13.7)	46 (22.9)	0.046
Symptomatic	4 (3.2)	20 (9.3)	0.033	3 (2.6)	18 (9)	0.027
Median time to any HT, days (IQR)	1 (1–1)	1 (1–1)	0.763	1 (1–1)	1 (1–1)	0.608
Any HT occurred within 24 h after IVT, *n* (%)	17 (13.5)	44 (20.5)	0.105	13 (11.1)	40 (19.9)	0.043
**Secondary**						
NIHSS (M, IQR)						
Day 1, all patients	5 (3–11)	6 (3–11)	0.536	5 (3–9)	5 (3–10)	0.412
Day 1, patients with any HT	11 (5–16)	14 (6–17)	0.493	8 (5–13)	14 (8–17)	0.158
Day 14, all patients	2 (1–6)	3 (2–7)	0.045	2 (1–6)	3 (1–6)	0.032
Day 14, patients with any HT	5 (2–11)	7 (2–13)	0.370	5 (2–8)	7 (2–12)	0.183
mRS, day 90 (M, IQR)	1 (0–2)	1 (1–3)	0.240	1 (0–2)	1 (0–3)	0.148
Favorable outcome, *n* (%)	95 (75.4)	150 (69.8)	0.265	95 (81.2)	150 (74.6)	0.179

Likewise, Cerebrolysin treatment resulted in a substantial decrease of symptomatic HT (ITT population: 3.2% compared to 9.3%; PP population: 2.6% compared to 9.0%) with an OR of 0.248 (95% CI: 0.072–0.851; *p* = 0.019) and 0.171 (95% CI: 0.040–0.726; *p* = 0.022), respectively.

In the ITT population, the NNT (benefit) to reduce any and symptomatic HT with Cerebrolysin was 13.545 (95% CI, 68.282 (harm) to 6.161 (benefit)) and 16.319 (95% CI, 8.536 (benefit) to 184.973 (benefit)), respectively.

Similarly, in the PP cohort, the NNT (benefit) was 10.86 (95% CI, 5.50 (benefit) to 420.68 (benefit)) and 15.65 (95% CI, 8.33 (benefit) to 129.10 (benefit)), respectively.

**Fig. 3:**
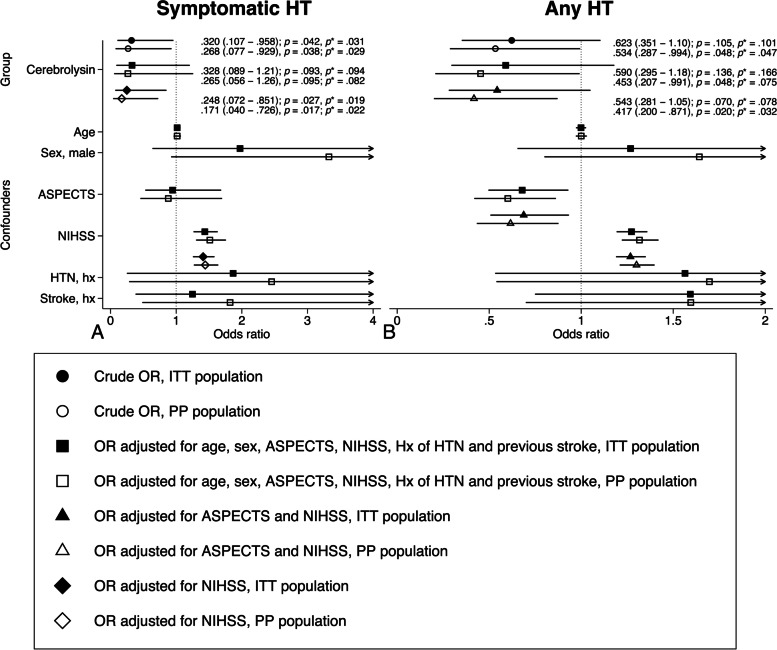
Primary endpoints. Univariate and multivariate logistic regression. Crude and adjusted OR with CI are reported. Solid markers – intention-to-treat population (*n* = 341, Cerebrolysin = 126, Control = 215); hollow markers – per-protocol population (*n* = 318, Cerebrolysin = 117, Control = 201). Circles – crude OR; squares – OR adjusted for age, sex, ASPECTS, NIHSS, history of hypertension and previous stroke; triangles – OR adjusted for ASPECTS and NIHSS; diamonds – OR adjusted for NIHSS; p* – Romano–Wolf adjusted *p*-values. **A.** Symptomatic HT. **B.** Any HT

Although the arms were imbalanced in age, history of hypertension and previous stroke, the differences had no effect on the primary endpoints after adjustment in a multivariate logistic regression model. In fact, the NIHSS and ASPECTS were determined as significant confounders for any HT, while symptomatic HT was confounded by the NIHSS (Fig. 3).

### Secondary endpoints

**Fig. 4:**
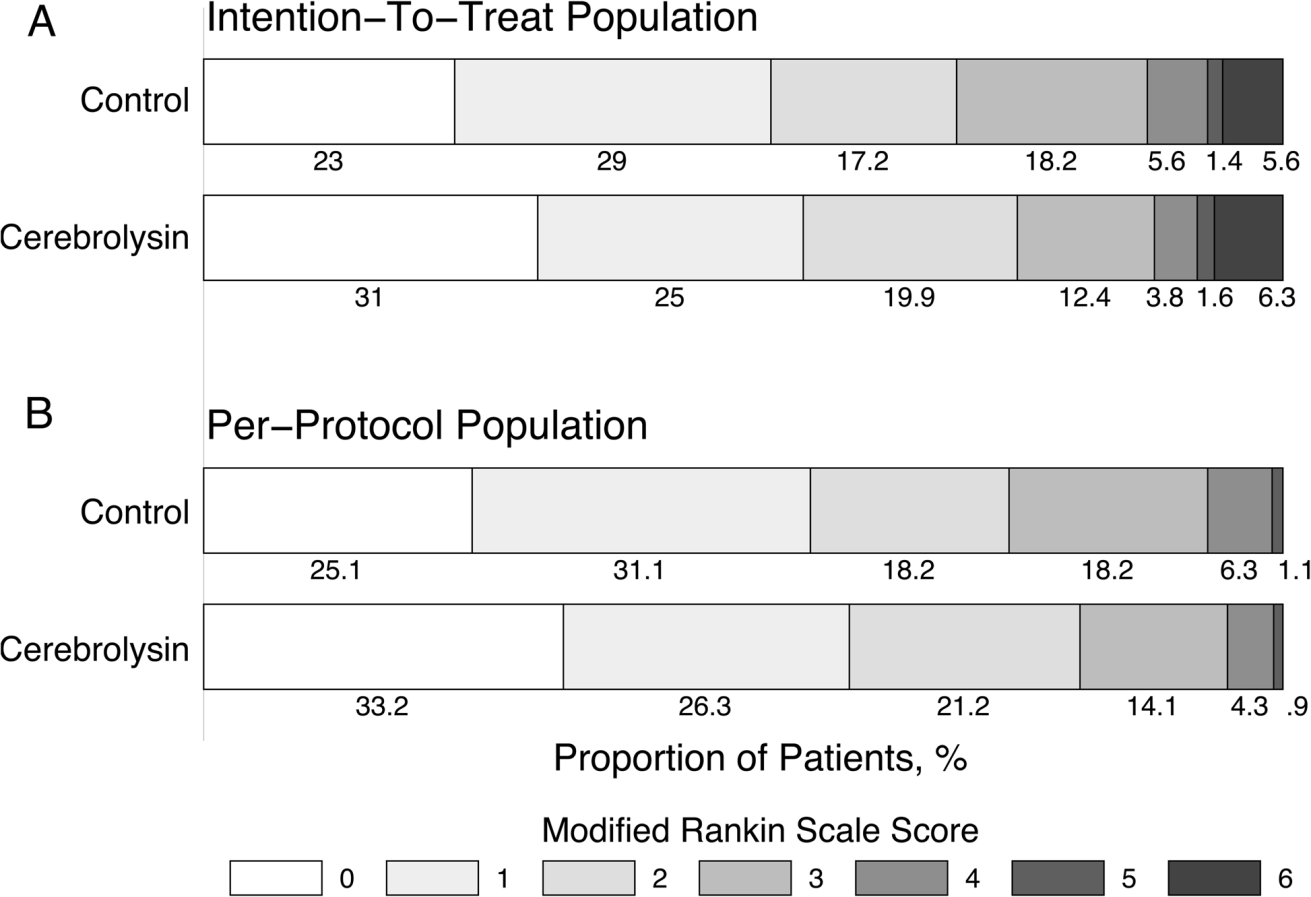
Secondary endpoint. Modified Rankin Scale score on day 90. **A.** Intention-to-treat population (*n* = 341, Cerebrolysin = 126, Control = 215). **B.** Per-protocol population (*n* = 318, Cerebrolysin = 117, Control = 201)

The percentage of patients with a favorable functional outcome was approximately the same in both groups. Early neurological recovery on day 14 was more noticeable in the Cerebrolysin group. However, the difference disappeared in patients with HT (Table 5, Fig. 4).

No serious AE related to Cerebrolysin were observed. Several mild-to-moderate AE occurred in both groups within 48 h after treatment initiation and lasted up to a few hours without any consequences. The study investigators had unanimously agreed that none of them were attributed to the studied medication, except mild agitation, a well-known side effect of Cerebrolysin (Table 6).

**Table 6:** Safety analysis

AE, *n* (%)	ITT Population			PP Population		
	**Cerebrolysin** **, ** ***n*** ** = 126**	**Control, ** ***n*** ** = 215**	***p*** **-Value**	**Cerebrolysin** **, ** ***n*** ** = 117**	**Control, ** ***n*** ** = 201**	***p*** **-Value**
Hypotension	1 (0.8)	1 (0.5)	0.701	1 (0.9)	1 (0.5)	1.000
Fever	4 (3.2)	2 (0.9)	0.128	4 (3.4)	2 (1)	0.198
Headache	1 (0.8)	3 (1.4)	0.618	0 (0)	3 (1.5)	0.300
Agitation	4 (3.2)	1 (0.5)	0.045	3 (2.6)	0 (0)	0.049
Nausea, vomiting	2 (1.6)	1 (0.5)	0.284	2 (1.7)	1 (0.5)	0.557
Other gastrointestinal symptoms	1 (0.8)	1 (0.5)	0.701	1 (0.9)	1 (0.5)	1.000
Nuisance bleeding	1 (0.8)	1 (0.5)	0.701	1 (0.9)	1 (0.5)	1.000
Death	8 (6.3)	12 (5.6)	0.771			
Symptomatic HT	1 (0.8)	2 (0.9)	0.798			
Malignant brain edema	3 (2.4)	5 (2.3)	0.974			
Myocardial infarction	2 (1.6)	3 (1.4)	0.887			
Pulmonary embolism	1 (0.8)	1 (0.5)	0.701			
Pneumonia	1 (0.8)	1 (0.5)	0.701			
Neurosurgery	1 (0.8)	1 (0.5)	0.701			
Life-threatening medical condition	0 (0)	1 (0.5)	0.443			

From day 0 to 14, there were no clinically significant abnormalities in the vital signs and laboratory tests in both groups. Overall, no safety concerns were noted for the concomitant use of IVT and Cerebrolysin.

### Advanced brain imaging

A total number of patients included in the advanced brain imaging analysis was 33 (Fig. 2). At baseline, characteristics of the groups were similar (Table 7).

**Table 7:** Baseline characteristics (*n* = 33), advanced brain imaging study

	**Cerebrolysin** **, ** ***n*** ** = 16**	**Control, ** ***n*** ** = 17**	***p*** **-Value**
Age, yr (M, IQR)	64.5 (55–77.8)	64 (55–70.5)	0.901
NIHSS (M, IQR)			
Admission	11 (6–14)	7 (5–12)	0.168
Day 1	5 (3–9)	5 (2–8)	0.557
Day 14	2 (1–6)	2 (2–4)	0.986
ASPECTS at admission (M, IQR)	10 (10–10)	10 (10–10)	0.817
Atrial fibrillation, *n* (%)	6 (37.5)	3 (18.8)	0.238
Diabetes mellitus, *n* (%)	1 (6.3)	3 (18.8)	0.285
Sex, male, *n* (%)	8 (50)	10 (58.8)	0.611
Weight, kg (M, IQR)	80 (65.5–90.5)	79 (65–93)	0.929
Systolic blood pressure, mm Hg (M, IQR)	155 (130–168.8)	150 (140–165)	0.606
Diastolic blood pressure, mm Hg (M, IQR)	90 (82.5–100)	90 (80–100)	0.790
Previous use of aspirin or antiplatelet drugs, *n* (%)	8 (50)	9 (52.9)	0.866
Hypertension, *n* (%)	12 (75)	15 (88.2)	0.325
History of stroke, *n* (%)	2 (12.5)	5 (31.3)	0.200
Door-to-needle time, min (M, IQR)	40 (31.3–40)	40 (30–42.5)	0.817
Stroke subtype, *n* (%)			
Atherothrombotic	2 (12.5)	6 (35.3)	0.127
Cardioembolic	9 (56.3)	6 (35.3)	0.227
Unknown etiology	5 (31.3)	5 (29.4)	0.909

No differences in the DTI metrics and DWI infarct volume between the groups were observed on day 1 (Table 8).

**Table 8:** DTI data, day 1

	**Cerebrolysin, ** ***n*** ** = 16**	**Control, ** ***n*** ** = 17**	***p*** **-Value**
AD, × 10^–6^ mm^2^/s (M, IQR)			
Affected side	586 (483.5–605)	602 (487.5–658)	0.581
Contralateral side	1116.5 (971.25–1174.5)	1169 (868.5–1337)	0.402
Laterality index, %	30.5 (20.25–40.25)	31 (25–42.5)	0.683
RD, × 10^–6^ mm^2^/s (M, IQR)			
Affected side	749 (593.3–919.5)	663 (579–753.5)	0.276
Contralateral side	1200.5 (1083.3–1414)	1095 (740.5–1274)	0.068
Laterality index, %	26.5 (15.5–37.3)	26 (13–35)	0.845
MD, × 10^–6^ mm^2^/s (M, IQR)			
Affected side	758 (671–801)	640 (492.5–772.5)	0.118
Contralateral side	1133 (889–1413)	1047 (813–1260.5)	0.423
Laterality index, %	23.5 (9–38.5)	25 (6.5–39.5)	0.929
FA, × 10^–4^ (M, IQR)			
Affected side	1975.5 (1777.3–2632.8)	2096 (1778–2390.5)	0.873
Contralateral side	3371 (2769.8–3792.3)	3574 (3409.5–3904.5)	0.053
Laterality index, %	25.5 (12.3–35.8)	28 (17.5–37)	0.790
DWI lesion volume, mL (M, IQR)	38.2 (29.3–53.9)	35.3 (23.6–52)	0.557

**Fig. 5:**
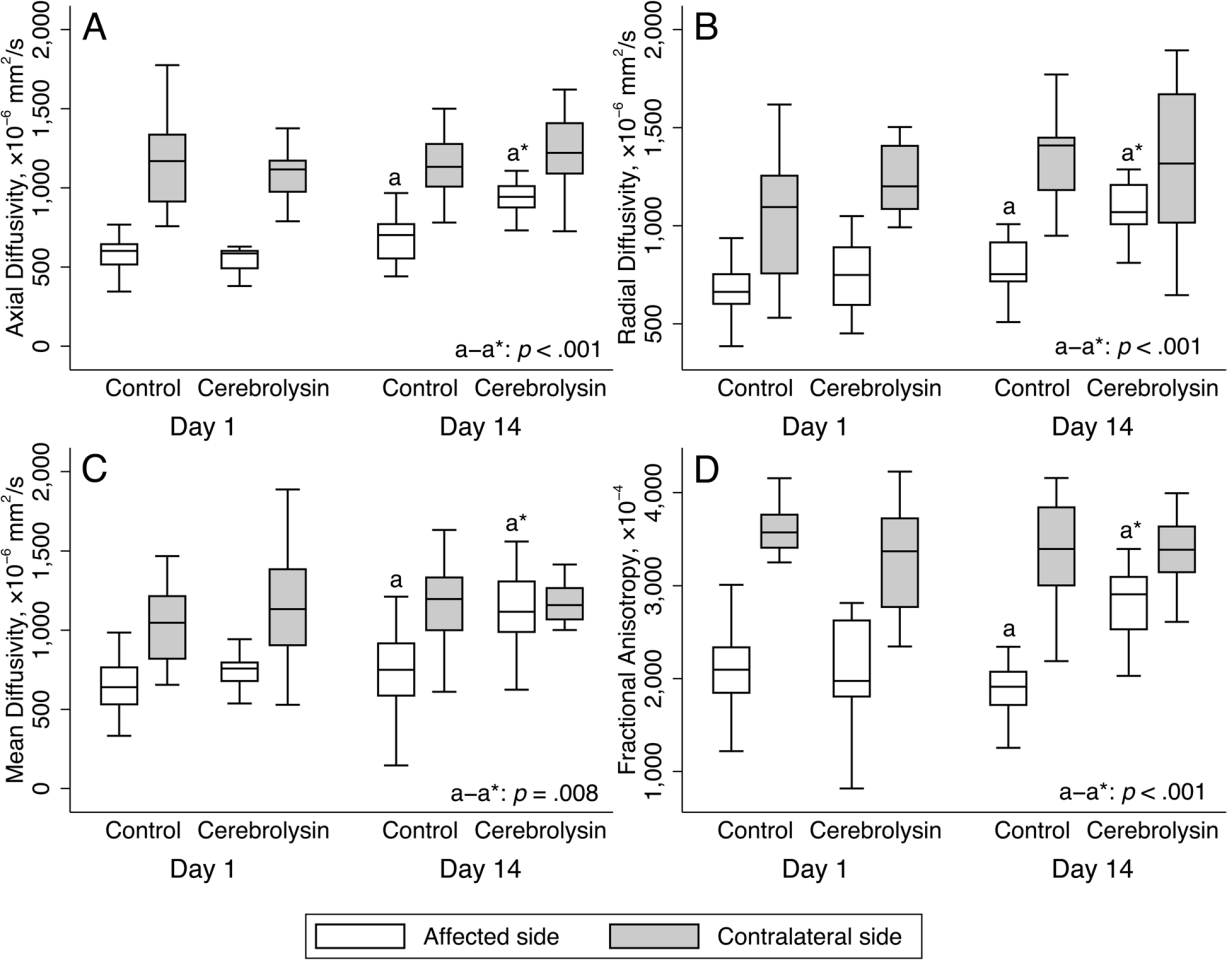
Advanced brain imaging analysis: diffusion-tensor imaging data (*n* = 33, Cerebrolysin = 16, Control = 17). Outliers are excluded; *p*-values of ≥ 0.05 are omitted. White boxes – affected side; gray boxes – contralateral side. **A.** Axial diffusivity, × 10^–6^ mm^2^/s. **B.** Radial diffusivity, × 10^–6^ mm^2^/s. **C.** Mean diffusivity, × 10^–6^ mm^2^/s. **D.** Fractional anisotropy, × 10^–4^. On the affected side, there was no difference between the arms in the DTI metrics on day 1. On day 14, *p*-values for pairwise comparison are reported on the affected side. On day 1 and 14, there was no difference between the groups on the contralateral side

However, patients treated with Cerebrolysin showed a significant improvement of their DTI data on day 14 (Table 9, Fig. 5).

**Table 9:** BBB permeability and DTI data, day 14

	**Cerebrolysin, ** ***n*** ** = 16**	**Control, ** ***n*** ** = 17**	***p*** **-Value**
AD, × 10^–6^ mm^2^/s (M, IQR)			
Affected side	943 (873.8–1033)	702 (544–776.5)	< 0.001
Contralateral side	1221 (1087.8–1420.8)	1133 (995–1299)	0.245
Laterality index, %	11.5 (6.7–20.3)	27.9 (13.8–36)	0.008
RD, × 10^–6^ mm^2^/s (M, IQR)			
Affected side	1069 (1004.3–1212)	753 (709.5–922)	< 0.001
Contralateral side	1317 (982.5–1700)	1409 (1175–1477)	0.631
Laterality index, %	15.2 (8–27)	26.6 (17.6–34.7)	0.025
MD, × 10^–6^ mm^2^/s (M, IQR)			
Affected side	1116.5 (964.5–1318)	750 (576–1046.5)	0.008
Contralateral side	1158.5 (1051–1281.5)	1197 (961–1362)	0.958
Laterality index, %	8.5 (4–17.8)	23 (15.5–36)	0.002
FA, × 10^–4^ (M, IQR)			
Affected side	2908 (2524.5–3113.5)	1913 (1628.5–2131)	< 0.001
Contralateral side	3387.5 (3136–3718.5)	3396 (2978.5–3915.5)	0.817
Laterality index, %	8.5 (6–15.8)	26 (20.5–37.5)	< 0.001
PS, mL/100 g/min (M, IQR)			
Affected side	1.24 (0.95–1.61)	2.46 (2.16–2.89)	< 0.001
Contralateral side	0.47 (0.36–0.6)	0.43 (0.3–0.53)	0.217
Laterality index, %	44.5 (29.3–58.8)	72 (64–78)	< 0.001
CT lesion volume, mL (M, IQR)	21.5 (15.9–26.6)	38.4 (30.9–42.5)	< 0.001

**Fig. 6:**
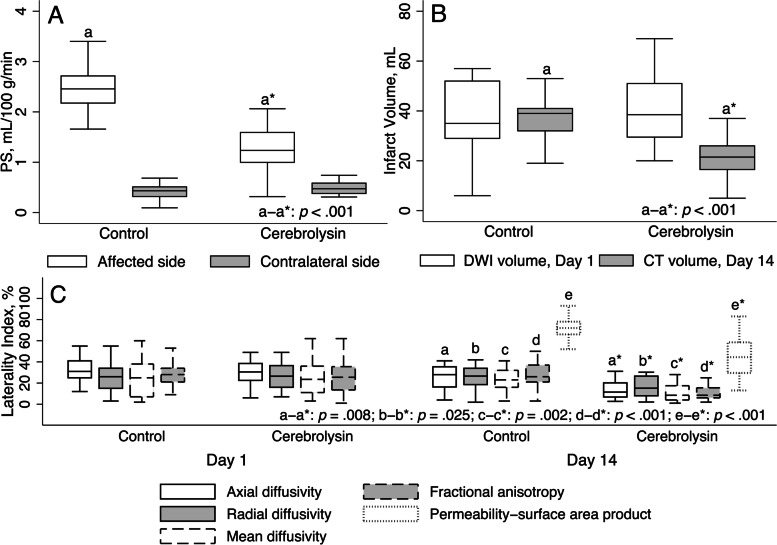
Advanced brain imaging analysis: permeability–surface area product, infarct volume, and laterality index (*n* = 33, Cerebrolysin = 16, Control = 17). Outliers are excluded; *p*-values of ≥ 0.05 are omitted. **A.** Permeability–surface area product, mL/100 g/min, day 14. White boxes – affected side; gray boxes – contralateral side. *p*-Values for pairwise comparison are reported on the affected side. No difference between the groups was found on the contralateral side. **B.** Infarct volume, mL. White boxes – DWI infarct volume, day 1; gray boxes – CT infarct volume, day 14. On day 14, *p*-values for pairwise comparison are reported. On day 1, there was no difference between the arms. **C.** Laterality index, %. Solid white boxes – axial diffusivity; solid gray boxes – radial diffusivity; dashed white boxes – mean diffusivity; dashed gray boxes – fractional anisotropy; dotted white boxes – permeability–surface area product. On day 14, *p*-values for pairwise comparison are reported. No difference between the groups was found on day 1

Moreover, the two-week treatment course with Cerebrolysin reduced the BBB permeability and CT infarct volume by more than 1.5-fold (Fig. 6).

## Discussion

The results of this randomized, open-label, multicenter pilot trial in stroke patients demonstrate beneficial effects of Cerebrolysin as an early add-on to IVT on the primary (the rate of symptomatic HT) and secondary (early neurological recovery) endpoints. Although we found a significant improvement in the DTI and PS values of the infarcted area, this treatment approach did not affect the long-term functional outcome.

A variety of composite scores to predict HT in AIS patients have been published [20]. Yet, pharmacological prevention of HT is still underdeveloped. Several pilot clinical studies have investigated the concomitant use of various agents, which reduce HT and exert multimodal effects, alongside rtPA. However, phase III clinical trials are required to confirm the observed positive results [21].

The risk of HT is established to be the highest on day 1 after stroke onset, and it keeps being significant for the next 14 days [22]. Consequently, pharmacological prevention of HT should be started as early as possible and should be continued for as long as 2 weeks.

Cerebrolysin treatment was started simultaneously with IVT and continued for 2 weeks. This approach seems to be safe and could alleviate symptomatic intracranial hemorrhagic events and early neurological deficit. Moreover, the beneficial effects correlated with the positive changes in the imaging metrics, supporting the neuroprotective and BBB stabilizing activity of Cerebrolysin. Thus, our findings are strongly coherent with the initial rationale.

However, the rate of symptomatic HT observed in our study was higher than it was reported in other IVT clinical trials [1, 16]. The difference could be attributed to a peculiar set of HT predictors in the recruited patients (Table 4) and required a separate sub-analysis beyond the scope of the current research.

In a prospective open-label study of AIS patients with futile recanalization after rtPA, Poljakovic et al. have demonstrated a clear trend towards HT rate reduction in patients treated with Cerebrolysin [23]. Although their Cerebrolysin course was initiated after rtPA, our findings have not contradicted their results. Another ongoing clinical trial, the efficacy of Cerebrolysin treatment as an add-on therapy to mechanical thrombectomy in patients with AIS due to large vessel occlusion, has set the rate of symptomatic HT as a secondary endpoint [24].

In a previous trial by Lang et al. (CERE-LYSE-1), the authors looked at the safety of Cerebrolysin administered 1 h after rtPA infusion for 10 consecutive days as well as at short- and long-term functional outcomes in AIS patients, but not at HT [4]. The results of our safety analysis are in good agreement with their data.

While our data on short-term neurological recovery confirms the results of previous trials [4, 5], we were not able to demonstrate a difference between the two groups in the long-term functional outcome. A larger sample size and shift analysis may be required to detect the effect [25] as well as a longer treatment course with Cerebrolysin.

The ESCAPE-NA1 trial, a large-scale study of the neuroprotective agent nerinetide, failed to demonstrate an improvement in the long-term post-stroke functional outcome due to a possible drug-drug interaction with alteplase [26]. Based on our results, Cerebrolysin could present an alternative treatment for such patients.

AD and RD could serve as in vivo surrogate markers of axonal and myelin damage, respectively [27]. FA is considered as an integrative indicator of the brain microarchitecture [28]. DTI alterations in the infarcted area at the acute stage of stroke correlate strongly with the extent of ischemic injury to the white matter. An early increase in the DTI metrics following the initial drop is associated with favorable neurological recovery [28, 29]. A significant improvement in the DTI data in the Cerebrolysin arm on day 14 can likely be attributed to the neuroprotective properties of Cerebrolysin since it consists of low-molecular weight neuropeptides and free amino acids, which mimics the action of endogenous neurotrophic factors on brain protection and repair [30]. Moreover, our findings are in line with previous data from patients with subacute ischemic stroke treated with Cerebrolysin [31].

PS is a known imaging marker of BBB permeability. The more PS rises following AIS, the higher the risk of HT [32]. The infarct volume on day 14 cannot be considered as final: it will have approached its finite dimensions by day 30 [33]. Hence, a dramatic reduction in the lesion size on day 14 in the Cerebrolysin group may be attributed to the attenuation of vasogenic edema due to BBB stabilizing features of Cerebrolysin. Our imaging results of BBB permeability support that assumption and are in accord with the experimental data [3].

The strength of the study came in simultaneous use of Cerebrolysin and rtPA followed by a combined assessment of clinical and advance brain imaging data in AIS patients, which has demonstrated multimodal effects of Cerebrolysin on brain recovery and HT prevention in the clinical settings.

However, our research had several limitations. It was not blinded, and standard medical care was applied to both groups.

Moreover, the sample size was relatively small. As a consequence, the imbalance in some covariates did occur which could be expected since exact balance is a large-sample property [34, 35]. The power calculation of the study was based on efficacy assumptions which proved to be not compatible for concomitant randomization of the imaging sub-study. Therefore, additional patient recruitment was required. As it was a pilot trial, we did not use sophisticated randomization procedure.

The study was also limited in terms of ethnic and racial diversity. The majority of the participants were of Russian, Tatar, and Jewish ethnic groups with no patients of African, Asian or Hispanic origin.

The analysis of DTI and CTP data was confined to a single slice. As a result, our assessment of the infarcted area was restricted. Moreover, our selection criteria for the advanced brain imaging could be a source of potential bias.

Therefore, additional large-scale clinical trials are warranted to confirm our findings.

## Conclusions

Early add-on of Cerebrolysin to reperfusion therapy was safe and significantly decreased the rate of symptomatic HT as well as early neurological deficit. However, no significant effect on day 90 functional outcome was detected. Improvements in the imaging metrics of the infarcted area support the neuroprotective and BBB stabilizing activity of Cerebrolysin.

## Data Availability

The datasets acquired and analyzed for this study are available from the corresponding author upon reasonable request.

## Abbreviations

AD: Axial diffusivity
AE: Adverse events
AIS: Acute ischemic stroke
ASPECTS: Alberta stroke program early CT score
BBB: Blood–brain barrier
CI: Confidence intervals
CT: Computed tomography
CTP: Computed tomography perfusion
DTI: Diffusion-tensor imaging
DWI: Diffusion-weighted imaging
FA: Fractional anisotropy
HT: Hemorrhagic transformation
IQR: Interquartile range
ITT: Intention-to-treat
IVT: Intravenous thrombolysis
M: Median
MD: Mean diffusivity
MRI: Magnetic resonance imaging
mRS: Modified Rankin scale
NIHSS: National Institutes of Health Stroke Scale
NNT: Number needed to treat
OR: Odds ratio
PP: Per-protocol
PS: Permeability–surface area product
RD: Radial diffusivity
rtPA: Recombinant tissue plasminogen activator
